# An Analysis of Peripheral Neuropathy Symptom Characteristics in HIV

**DOI:** 10.47363/jmhc/2022(4)179

**Published:** 2022-01-28

**Authors:** Joyce K Anastasi, Bernadette Capili, Donald J McMahon

**Affiliations:** 1 PhD Independence Foundation Endowed Professor Founding Director, Special Studies in Symptom Management New York University, New York, NY, USA; 2 PhD Rockefeller University Director, Heilbrunn Family Center for Research Nursing, New York, NY, USA; 3 Special Studies in Symptom Management Primary Statistician New York, NY, USA

**Keywords:** Pain, Neuropathy, HIV, Distal Sensory Peripheral Neuropathy, Symptom Characteristics, Symptom Science

## Abstract

**Objectives:**

A gap remains in understanding the association among the symptoms of distal sensory peripheral neuropathy (DSPN) (pain, aching, burning, pins and needles, numbness), comorbidities, and medication use among persons living with People Living with HIV/AIDS (PLWH) with DSPN. This report describes the symptom characteristics associated with prescribed treatment regimens (HIV and non-HIV medications) and comorbidities from a cohort of PLWH experiencing symptoms of DSPN who reside in New York City.

**Methods:**

Our sample (n=353) included PLWH who were 18 years or older, and with painful lower limb (LL) peripheral neuropathy screened for an ongoing clinical trial to reduce DSPN symptoms using acupuncture/moxibustion. The trial participants completed a screening interview where they reported age, gender, race, ethnicity, HIV status, presence of LL DSPN and DSPN symptoms, current medications, and comorbidities.

**Results:**

Of 465 persons screened, 353 provided information for inclusion in this analysis. Seventy-eight percent rated their LL DSPN in the “*severe*” or “*very severe*” discomfort/pain range. Nearly half of those were taking prescribed or over-the-counter medication, such as nonnarcotic analgesics, antidepressants, and anticonvulsants, to manage their DSPN discomfort/pain. Despite the use of OTC and or prescription pain relievers, participants reported insufficient symptom relief.

**Discussion:**

Combination Antiretroviral Therapies (CART) effectively control viral load and maintain healthy T-cell levels in individuals with HIV. It has made HIV a chronic disease for many. However, HIV DPSN remains prevalent and has a negative impact on the lives of PLWH. Our findings highlight that, despite the availability and the use of CART, DSPN remains prevalent and not well managed. A critical need exists for the development of effective interventions to manage DSPN symptoms.

## Introduction

Peripheral neuropathy (PN) is the most common neurological complication of HIV. It is characterized by debilitating pain, aching, burning, pins and needles, and numbness of the lower extremities and is estimated to affect 30% to 60% of PLWH [1]. PN is associated with lower scores on daily functional assessments. The causes of PN include HIV itself, comorbidities, and the multiple medications prescribed, including antiretroviral therapies (ART) [1,2]. Additional factors contributing to PN include vitamin C insufficiency and increased alcohol use [2].

The Foundation for Peripheral Neuropathy reports that approximately 33% of PLWH have some form of nerve impairment [2]. PN is also more common among older PLWH [2,3], and they are at greater risk for falling due to numbness, decreased sensitivity to touch, and muscle weakness which can affect balance.

This report was prepared because during our recruitment efforts for an HIV peripheral neuropathy study, we noticed that nearly half of potential participants were taking medications to manage the pain associated with PN but still reported high levels of pain and discomfort. Additionally, studies have demonstrated that women are at increased risk for chronic and greater pain severity, and racial disparities exist in pain management in the United States.

Considering the pain levels described by our potential participants and the published literature on women and race, we decided to explore the associations between PN symptoms, HIV, and non-HIV medications, gender, and race in our sample of PLWH who had LL PN (n=353).

## Methods

The study participants included individuals who called the New York University’s Division of Special Studies in Symptom Management to determine eligibility for an ongoing intervention study to reduce symptoms associated with PN. Potential participants completed a screening telephone interview conducted by trained research personnel. The screening objective was to recruit PLWH for an ongoing clinical trial titled: “*Symptom Management Efficacy Study to Reduce Distal Neuropathic Pain*” [Parent study - NIH-R01-NR017917]. The parent study is designed to evaluate the efficacy of acupuncture and a Chinese herbal medicine therapy known as moxibustion to reduce *moderate* or greater LL pain associated with PN. Full Institutional Review Board approval was received and registered in ClinicalTrials.gov NCT03855111. Data for this report was collected from January 2019 to March 2020.

Multiple approaches were used to recruit participants for the treatment study. These methods included advertisements in complimentary daily New York City-based newspapers and information packets sent to local health care institutions specializing in HIV treatment. The information packets included flyers about our research, study site location, contact information, informational brochures, and community outreach.

Potential participants called our research center, where trained research personnel conducted a structured screening interview to establish study eligibility. The interview included obtaining information on demographic characteristics, HIV status, presence of LL DSPN and DSPN symptoms, current medications, and comorbidities. For this report, the sample cohort included PLWH who were 18 years or older and self-identified as having painful LL DSNP. While 475 individuals inquired about the parent study, we are reporting on the 353 individuals who provided age, gender, race, and ethnicity.

## HIV/Neuropathy Response Measures

### HIV Status

An HIV+ diagnosis was determined by a ‘yes’ or ‘no’ response and an additional question, namely, “*when were you diagnosed with HIV*?” The HIV diagnosis date was classified as a categorical variable with a specific year range. This analysis classified the diagnosis date into six categories using the year 2020 as the designated reference date. These categories included <2 years, 2–5 years, 5–10 years, 10–15 years, 15–20 years, and >20 years.

### Neuropathy Status

The presence of DSPN was determined by a ‘yes’ or ‘no’ response and an additional question “*Are you currently experiencing neuropathy in your feet, legs, or both*?” To further assess DSPN pain, the analysis incorporated a seven-point Likert scale to determine the severity of pain. The Likert scale used in this analysis ranged from 0–6 with 0 indicating ‘*none*’ or no pain, 1 ‘*minimal*’, 2 ‘*mild*’, 3’ *moderate*’, 4’ *moderately severe*’, 5 ‘*severe*’ and 6, representing the highest intensity level of pain identified as ‘very severe.’ Responses to the pain scale were incorporated into this analysis as a continuous variable.

Additionally, we collected the length of time potential candidates experienced DSPN pain. The response options were coded as either > 3 months or < 3 months. The response question was asked directly after asking candidates if they were currently experiencing DSPN with a follow-up question of “*how long*?”.

## Outcome Measures

### Medication Classifications

The team members reviewed all medication responses and categorized medication classes. These medication classes were grouped into three categories: HIV, neuropathy pain and medications, and non-HIV medications.

### HIV Medications

HIV medications were defined using the “Positively Aware HIV Drug Chart” sponsored by the “Test Positive Aware Network” (TPAN) [4]. TPAN is an organization of expert HIV professionals that aims to provide HIV-related health information, including medication guides [4]. The HIV medications were defined categorically as single-tablet regimens (STR), integrase inhibitors (INSTI), entry inhibitors (EI), nucleotide reverse transcriptase inhibitors (NRTI), non-nucleotide reverse transcriptase inhibitors (NNRTI), protease inhibitors (PI), pharmacokinetic enhancers (PKE), as well as legacy drugs (LD) (medications no longer or rarely prescribed).

### Neuropathy Medication

Neuropathy medication categories were defined and grouped based on published literature [1,5]. For instance, Morris and McCarberg evaluated antidepressants and antiepileptic medications used for pain management in non-cancer patients [6]. Through their research, antiepileptics, as well as antidepressant analgesics used in this study, were easily defined; and categorized based on select candidate responses. These medications included gabapentin (antiepileptic), and amitriptyline (antidepressant). Additional information on the literature used to classify the samples of neuropathy medications is detailed elsewhere [1,5].

Candidates were asked, “*Are you currently taking pain medications for your peripheral neuropathy? If you are, please name these medications*.” Responses were reviewed and categorized into one of the following categories: antiepileptic analgesics, nonsteroidal anti-inflammatory drugs (NSAIDs), and other, which include acetaminophen, antidepressant-analgesics, and opioids. Rarely used medications were categorized into the ‘other’ category (e.g., lidocaine patches and marijuana).

### Non-HIV Medications

All medications that were not HIV or DSPN related were grouped into disease-specific categories based on the WHO’s International Classification of Diseases Revision 11 (ICD-11). These medication categories included psych-mental health, cardiovascular, diabetes mellitus, respiratory (asthma), and other. The medication created groups derived from the ICD-11 included prescribed and over-the-counter drugs.

### Neuropathy Symptoms

DSPN symptom location was coded as only feet, feet, and legs, or other. Symptom quality was assessed as ‘aching,’ ‘pins and needles,’ ‘burning,’ and ‘numbness.’

### Statistical Approach

Data were summarized with counts and percent of the group for categorical items and means and medians for continuous variables. Cross-tabulations were used to examine demographic, LL DSPN pain severity and deficit quality, HIV and non-HIV medication use, medications for neuropathic pain. We did not test all comparisons between or among subsets of the sample as our goals are primarily descriptive. We used Fisher’s Exact Test where subset proportions appeared substantially different. Data were analyzed using SAS software Version 9.4 for Windows.

## Results

### Sample Characteristics

Three hundred and fifty-three potential participants provided data. Table 1 shows the breakdown of demographics, duration of HIV disease, LL DSPN with current symptoms, rating of LL DSPN pain severity, and the qualitative categories for these neuropathy symptoms by age, gender, and race. The majority of the sample was male (62%), over age 50 (78%), and either Black/African American or other non-white race (79%). Nearly 70% were diagnosed with HIV more than 20 years ago. Ninety-two percent reported current LL DSPN symptoms, again evenly distributed across age, gender, and race categories. Potential participants rated the severity of their discomfort/pain on a seven-point scale from 0 to 6. Seventy-four percent reported pain severity in the most severe two levels of the scale, with women reporting more severe levels than men (p<0.001) and Black/African Americans more than whites (p<0.03); however, pain severity appeared similarly distributed in the age under and over 50 subgroups(p<0.83). About three-quarters of the sample endorsed each of the four neuropathy symptom quality categories. All candidates endorsed sensations of pins and needles, and numbness more frequently than aching or burning.

### Demographics of HIV medication use, non-HIV medication use, and comorbidities, medications, and treatments for neuropathic pain

The cohort is described by demographics, age, under and over age 50, gender and race, and their current regimen of HIV medications; non-HIV medications or the comorbidity implied by the medication they are taking; and medications taken for neuropathic symptom relief.

Table 2. Ninety-five percent are taking at least one medication for HIV. STRs are most common (64%) followed by NRTIs (23%). Persons in the older age category tended to more frequently be taking NRTIs relative to those in the younger age category(p<0.08), who more frequently are taking STRs(p<0.03). Men and women present similarly in these regimens. Black/African Americans were less frequently taking NRTIs(p<0.02). No other demographic group differences were found.

Non-HIV medications mentioned, and comorbidities identified, with or without an associated medication, were summarized with those categories with 5% or fewer mentions aggregated into an “Other” category. The drugs and comorbidities with 5% or fewer mentions in the cohort accumulated to represent 46% of the cohort. Only cardiovascular-related drugs and conditions elicited a similar proportion from the cohort: 38%. Seventy-three percent were taking at least one non-HIV medication. About 45% of persons in both age categories were taking at least one drug, and cardiovascular-related medications were more prevalent in the older age group(p<0.001). Respiratory and psychological/mood medication use was similar between age groups. Among men and women, the use of non-HIV medications as similar at about 74%, and gender imbalances in specific medication use were not seen. Among the races, whites were more frequently taking at least one of these medications with psychological/mood-related medications with common(p<0.01). Forty six percent of the sample were taking medicines for neuropathic symptom relief. This proportion did not differ by age, gender, or race categories. Antiepileptic drugs were the most common class of drugs prescribed for this purpose.

### HIV medication use, non-HIV medication use, comorbidities, medications and treatments for neuropathic pain and polypharmacy relation to Lower Limb Peripheral Neuropathy Symptoms

Table 3 repeats the categories of HIV medications, non-HIV medications, and comorbidities, neuropathic pain relief meds and profiles of LL DSPN symptoms by pain/discomfort severity, the distribution of symptoms to feet or both feet and legs, and quantifies neuropathic symptom: aching, burning, pins and needles and numbness. Persons reporting taking an HIV medication had a higher average neuropathic pain/discomfort severity (5.14) than those few reporting taking no HIV medications (4.80), but only five reported taking no HIV medications. Since NNRTIs alone, post-attachment inhibitors, legacy drugs, and entry inhibitors were rarely used, there are too few counts to suggest symptom pattern differences with these drugs.

Among the remaining HIV medication classes, neuropathic symptoms were most prevalent in those taking STRs (64%), over twice that of any other drug class. Numbness and burning sensations may be more common than aching or pins and needles for persons taking NRTIs. STRs followed by NRTIs associated with all four sensations, about 65% and 23%, respectively. None of the HIV medications showed a pattern of more frequent distribution in the feet alone than in the broader feet plus legs category. Among non-HIV medications and comorbidities, those endorsing none of these reported pain/discomfort severity levels no different from those taking any of these, although 73% taking any reported symptoms while only 21% of those taking none reported LL neuropathic symptoms. Neither those with cardiovascular- or diabetes-related conditions were more likely to report symptoms with distribution limited to the feet. Persons taking any medications for neuropathic pain relief (46% of cohort) reported higher pain/discomfort severity than persons taking none, p<0.02, and persons taking NSAIDs reported the highest average severity ratings, but this did not attain statistical significance (p<0.10). Symptom’s type or distribution between feet or feet plus legs did not differ significantly by the neuropathic pain relief medication category.

## Discussion

Historically, HIV DSPN was associated with the use of first-generation HIV medications with recognized neuro-toxic side effects i.e., zalcitabine, didanosine, and stavudine. While current CART effectively controls viral loads and maintains healthy T-cell levels, HIV DPSN remains prevalent and negatively impacts the lives of PLWH. From the 465 persons requesting information for entry into the clinical trial, 353 provided age, sex, race/ethnicity information for inclusion in this paper, 78% rated their LL DSPN in the “*severe*” or “*very severe*” discomfort/pain range. Nearly half were taking prescribed or over-the-counter medication, such as nonnarcotic analgesics, antidepressants, and anticonvulsants, to manage their DSPN discomfort/pain. Despite this use, our cohort reported insufficient symptom relief. Currently, there are no FDA-approved agents to treat non-diabetic DSPN pain and related symptoms.

With the effective CART treatments, HIV has transitioned to a chronic condition with an aging population and increased frailty across the lifespan [7,8]. The risk of DSPN also rises with age. Comorbidities were prevalent in this cohort. Seventy-three percent reported a non-HIV disease or taking a drug for other medical conditions. Cardiovascular disease (CVD) was the most prevalent at 38%, which is similar to the rate reported in a non-HIV group, wherein CVD is the leading cause of death in the US and globally [9]. These findings are consistent with the literature that report rates of CVD have doubled from 2.0% to 4.6% in PLWH in the US [10]. HIV infection is also an independent risk factor for coronary artery disease and heart failure [10].

We found the data shown in Table 3 interesting. There was a higher proportion of persons taking STRs reporting LL DSPN symptoms than those taking any other HIV regimen; however, symptom quality frequencies were similar. Only five persons were not taking any HIV medications, therefore we could not assess whether HIV medication exposure is associated with DSPN symptoms. Additionally, 73% of the cohort with cardiovascular, diabetes-related, respiratory, or other comorbidities was anticipated given the older minority male profile of the group. Those reporting any comorbidity were nearly three times more likely to report LL DSPN symptoms than those without an identified comorbidity.

## Limitations

The cohort was self-selected, and candidates were applying to enroll in a study to treat symptomatic DSPN. The cohort provided self-report of the data, and the observational nature of this study limited our ability to make causal inferences. Viral load and CD4 counts were not collected during the telephone screen, limiting the ability to evaluate associations with viral status and DSPN symptoms. However, it is essential to note that the prevalence of HIV-related DSPN reported in this study is similar to other HIV DSPN studies [11,12].

## Conclusion

This study describes the symptom experience with prescribed treatment regimens (HIV and non-HIV medications) and comorbidities from a cohort of PLWH experiencing symptoms of DPSN and living in New York City. The findings highlight the importance that, despite the availability and the use of CART, which has changed HIV to be a chronic condition, *DSPN remains prevalent and under-addressed*. Furthermore, DSPN in PWLH occurs in high-income countries, like the United States (US), and not just in low-income countries without adequate access to advanced HIV care. Therefore, a critical need exists for effective interventions to manage painful DSPN symptoms [13–23].

## Figures and Tables

**Table 1:** Demographic and Peripheral Neuropathy Symptom Characteristics: n (%)

Item	Coding	Total	Age		Gender		Race		
			Age < 50	Age 50>	Female	Male	Black/AA	White	Other
Age > 50		271(78)	76(100)	271(0)	113(86)	158(73)	186(78)	60(83)	25(69)
Gender	% Male	215(62)	57(75)	158(58)	0(0)	215(100)	136(57)	55(76)	24(67)
Ethnicity	% Hispanic	72(21)	19(25)	113(42)	27(20)	45(21)	26(11)	25(35)	21(58)
Race	% Non-white	275(79)	64(84)	211(78)	115(83)	160(74)	239(100)	0(0)	36(100)
Duration w/HIV	<2yrs	1(0)	1(1)	0(0)	0(0)	1(0)	1(0)	0(0)	0(0)
	2 – < 5 yrs	6(2)	3(4)	3(1)	1(1)	5(2)	3(1)	2(3)	1(3)
	5 – < 10 yrs	19(5)	9(12)	10(4)	5(4)	14(7)	16(7)	3(4)	0(0)
	10 – < 15 yrs	40(12)	19(25)	21(8)	14(11)	26(12)	29(12)	6(8)	5(14)
	15 – < 20 yrs	43(12)	18(24)	25(9)	16(12)	27(13)	27(11)	9(13)	7(19)
	>20yrs	238(69)	26(34)	212(78)	96(73)	142(66)	163(68)	52(72)	23(64)
Current LL PN Severity Rating (0 – 6)	n (%Yes)	319(92)	71(93)	248(92)	122(92)	197(92)	220(92)	66(92)	33(92)
	2	4(1)	1(1)	3(1)	1(1)	3(1)	3(1)	1(1)	0(0)
	3	18(5)	2(3)	16(6)	2(2)	16(7)	8(3)	8(11)	2(6)
	4	66(19)	13(17)	53(20)	17(13)	49(23)	49(21)	14(19)	3(8)
	5	98(28)	28(37)	70(26)	32(24)	66(31)	59(25)	24(33)	15(42)
	6	161(46)	32(42)	129(48)	80(61)	81(38)	120 (50)	25 (35)	16 (44)
Categories of LL PN									
Aching	n (%Yes)	254 (73)	58 (76)	196 (72)	95 (72)	159 (74)	180 (75)	51 (71)	23 (64)
Burning	n (%Yes)	232 (67)	53 (70)	179 (66)	88 (67)	144 (67)	164 (69)	45 (63)	23 (64)
Pins & Needles	n (%Yes)	290 (84)	58 (76)	232 (86)	108 (82)	182 (85)	199 (83)	63 (88)	28 (78)
Numbness	n (%Yes)	282 (81)	60 (79)	222 (82)	107 (81)	175 (81)	193 (81)	63 (88)	26 (72)

**Table 2:** Medication: HIV, non-HIV and comorbidities, neuro-active and polypharmacy. n (% Yes)

			Age		Gender		Race		
Item		Total	Age<50	Age50>	Female	Male	Black/AA	White	other
**HIV Meds**	Pls	59(17)	11(14)	47(17)	19(14)	39(18)	40(17)	9(13)	9(25)
	NRTIs	81(23)	12(16)	69(25)	26(20)	55(26)	47(20)	22(31)	12(33)
	NNRTis	12(3)	2(3)	10(4)	3(2)	9(4)	9(4)	2(3)	1(3)
	INSTis	62(18)	9(12)	53(20)	28(21)	34(16)	40(17)	15(21)	19(40)
	STRs	221(64)	57(75)	164(61)	87(66)	134(62)	154(65)	45(63)	21(58)
	Post-attachment inhibitors	20(6)	3(4)	17(6)	5(4)	15(7)	14(6)	3(4)	3(8)
	Legacy Drugs	6(2)	0(0)	6(2)	1(1)	5(2)	4(2)	2(3)	0(0)
	Entry inhibitors	5(1)	1(1)	4(1)	0(0)	5(2)	1(0)	1(1)	3(8)
	Other	21(6)	2(3)	19(7)	6(4)	15(7)	14(6)	5(7)	2(6)
	None	5(1)	1(1)	4(1)	2(2)	3(1)	5(2)	0(0)	0(0)
	Any HIV Med	330(95)	73(96)	257(95)	126(95)	204(95)	226(95)	70(97)	34(94)
**Non-HIV Meds and Comorbidities**	Psych	67(19)	14(18)	53(20)	19(14)	48(22)	37(15)	22(31)	8(22)
	CV related	131(38)	11(14)	120(44)	50(38)	81(38)	88(37)	31(43)	12(33)
	DM	30(9)	3(4)	27(10)	17(13)	13(6)	21(9)	6(8)	3(8)
	Respiratory	42(12)	8(11)	34(13)	20(15)	22(10)	29(12)	10(14)	3(8)
	Other	159(46)	33(43)	126(46)	66(50)	93(43)	102(43)	38(53)	19(53)
	None	74(21)	25(33)	49(18)	27(20)	47(22)	56(23)	11(15)	7(19)
	Any non-HIV Meds	255(73)	48(63)	207(76)	98(74)	157(73)	170(71)	59(82)	26(72)
**Neuropathy Meds**	Anti-epileptic	102(29)	18(24)	84(31)	44(33)	58(27)	65(27)	22(32)	14(39)
	NSAID	32(9)	9(12)	23(8)	13(10)	19(9)	25(10)	6(8)	1(3)
	Other	48(14)	12(16)	36(20)	20(15)	28(13)	32(13)	14(19)	2(6)
	Non	184(53)	43(57)	141(52)	65(49)	119(55)	132(55)	33(46)	19(53)
	Any Neuro Meds	159(46)	33(43)	126(46)	65(49)	94(44)	105(44)	38(53)	16(44)
**Polypharmacy (non-HIV Meds)**		29(8)	3(4)	26(10)	9(7)	20(9)	21(9)	6(8)	2(6)

**Table 3:** Symptom severity and distribution by Medications: HIV, non-HIV and comorbidities, neuro-active and polypharmacy. n (% Yes)

		LL PN Severity*	All	Aching		Burning		Pins & Needles		Numbness	
		Mean		Feet	Feet/Legs	Feet	Feet/Legs	Feet	Feet/Legs	Feet	Feet/Legs
HIV Meds	Pls	5.12	58(17)	19(22)	19(11)	21(19)	19(16)	21(15)	24(16)	28(19)	22(16)
	NRTIs	5.09	81(23)	20(23)	38(23)	29(26)	29(24)	30(21)	38(24)	36(25)	28(20)
	NNRTis	5.25	12(3)	6(7)	4(2)	7(6)	4(3)	6(4)	4(3)	5(3)	3(2)
	INSTIs	5.32	62(18)	15(17)	31(19)	21(19)	22(18)	19(14)	31(21)	28(19)	23(17)
	STRs	5.22	221(64)	59(67)	105(63)	73(65)	71(60)	94(67)	91(61)	93(65)	85(62)
	Post –attachment inhibitors	5.20	20(6)	7(8)	7(4)	7(6)	5(4)	4(3)	10(7)	9(6)	9(7)
	Legacy Drugs	5.33	6(2)	2(2)	4(2)	4(4)	1(1)	1(1)	5(3)	3(2)	1(1)
	Entry Inhibitors	5.00	5(1)	2(2)	1(1)	1(1)	1(1)	3(2)	0(0)	2(1)	2(1)
	other	4.95	21(6)	2(2)	12(7)	2(2)	12(19)	8(6)	12(8)	5(3)	14(10)
	None	4.80	5(1)	1(1)	2(1)	1(1)	1(1)	2(1)	1(1)	0(0)	2(1)
	Any HIV Med	5.14	330(95)	86(98)	157(95)	112(99)	111(93)	133(95)	142(95)	141(98)	128(93)
Non- HIV Meds Comorbidities	Psych	5.06	67(19)	17(19)	36(22)	24(21)	29(24)	28(20)	31(21)	28(19)	30(22)
	CV	5.11	131(38)	32(36)	61(37)	41(36)	35(29)	52(37)	60(40)	60(42)	44(32)
	DM	5.40	30(9)	4(5)	12(7)	14(12)	8(7)	13(9)	14(9)	20(14)	6(4)
	Respiratory	5.17	42(12)	10(11)	20(12)	15(13)	14(12)	20(14)	16(11)	20(14)	16(12)
	Other	5.06	159(46)	34(39)	81(49)	52(46)	54(45)	70(50)	65(43)	68(47)	64(46)
	None	5.19	74(21)	24(27)	31(19)	23(20)	28(24)	24(17)	31(21)	25(17)	31(22)
	Any Non- HIV Meds	5.11	255(73)	62(70)	124(75)	87(77)	81(68)	108(77)	109(73)	113(78)	98(71)
Neuropathy Meds	Anti-epileptic	5.21	102(29)	26(30)	53(32)	36(32)	38(32)	43(31)	46(31)	44(31)	45(33)
	NSAID	5.41	32(9)	9(10)	13(8)	8(7)	11(9)	7(5)	18(12)	11(8)	14(10)
	Other	5.36	48(14)	8(9)	28(17)	16(14)	19(16)	17(12)	27(18)	21(15)	21(15)
	None	5.03	184(53)	47(53)	82(49)	59(52)	60(50)	75(54)	73(49)	75(52)	69(50)
	Any Neuro Meds	5.27	159(46)	41(47)	82(49)	54(48)	56(47)	63(45)	76(51)	69(48)	67(49)
Polypharmacy (Non-HIV Meds)		5.17	29(8)	8(9)	12(7)	10(9)	10(8)	12(9)	12(8)	14(10)	9(7)

* Pain severity scale: 0- ‘*none*’ or no pain, 1 ‘*minimal*’, 2 ‘mild’, 3 ‘*moderate*’, 4 ‘*moderately severe*’, 5 ‘*severe*’ and 6, *very severe*.’
